# Oxidative Stress Is Associated with an Increased Antioxidant Defense in Elderly Subjects: A Multilevel Approach

**DOI:** 10.1371/journal.pone.0105881

**Published:** 2014-09-30

**Authors:** Gemma Flores-Mateo, Roberto Elosua, Teresa Rodriguez-Blanco, Josep Basora-Gallisà, Mònica Bulló, Jordi Salas-Salvadó, Miguel Ángel Martínez-González, Ramon Estruch, Dolores Corella, Montserrat Fitó, Miquel Fiol, Fernando Arós, Enrique Gómez-Gracia, Isaac Subirana, José Lapetra, Valentina Ruiz-Gutiérrez, Guillermo T. Sáez, Maria-Isabel Covas, for the PREDIMED Study Investigators

**Affiliations:** 1 Unitat de Suport a la Recerca Tarragona-Reus, Institut Universitari d′Investigació en Atenció Primària Jordi Gol (IDIAP Jordi Gol), Tarragona, Spain; 2 CIBER Fisiopatología Obesidad y Nutrición (CIBEROBN), Madrid, Spain; 3 Epidemiology and Cardiovascular Genetics Research Group, Institut Municipal d′Investigació Mèdica (IMIM), Barcelona, Spain; 4 CIBER de Epidemiología y Salud Pública (CIBERESP), Madrid, Spain; 5 Institut Universitari d′Investigació en Atenció Primària Jordi Gol (IDIAP Jordi Gol), Barcelona, Spain; 6 Human Nutrition Unit, School of Medicine, University Rovira i Virgili, Reus, Spain; 7 Department of Preventive Medicine and Public Health, University of Navarra-Osasunbidea, Servicio Navarro de Salud, Pamplona, Spain; 8 Department of Internal Medicine, Hospital Clinic, Institut dInvestigacions Biomèdiques August Pi Sunyer (IDIBAPS), Barcelona, Spain; 9 Department of Preventive Medicine and Public Health, University of Valencia, Valencia, Spain; 10 Cardiovascular Risk and Nutrition Research Unit, Institut Municipal d′Investigació Mèdica (IMIM), Barcelona, Spain; 11 Institute for Health Sciences Investigation, IdISPa, Palma de Mallorca, Spain; 12 Department of Cardiology, Hospital Txangorritxu, Vitoria, Spain; 13 Department of Epidemiology, School of Medicine of Malaga, Malaga, Spain; 14 Department of Family Medicine, Primary Care Division of Sevilla, San Pablo Health Center, Sevilla, Spain; 15 Instituto de la Grasa, CSIC, Sevilla, Spain; 16 Department of Biochemistry and Molecular Biology, Faculty of Medicine, Service of Clinical Analysis, Doctor Peset University Hospital, University of Valencia, Valencia, Spain; Instituto de Biociencias - Universidade de São Paulo, Brazil

## Abstract

**Background:**

Studies of associations between plasma GSH-Px activity and cardiovascular risk factors have been done in humans, and contradictory results have been reported. The aim of our study was to assess the association between the scavenger antioxidant enzyme glutathione peroxidase (GSH-Px) activity in plasma and the presence of novel and classical cardiovascular risk factors in elderly patients.

**Methods:**

We performed a cross-sectional study with baseline data from a subsample of the PREDIMED (PREvención con DIeta MEDiterránea) study in Spain. Participants were 1,060 asymptomatic subjects at high risk for cardiovascular disease (CVD), aged 55 to 80, selected from 8 primary health care centers (PHCCs). We assessed classical CVD risk factors, plasma oxidized low-density lipoproteins (ox-LDL), and glutathione peroxidase (GSH-Px) using multilevel statistical procedures.

**Results:**

Mean GSH-Px value was 612 U/L (SE: 12 U/L), with variation between PHCCs ranging from 549 to 674 U/L (Variance = 1013.5; *P*<0.001). Between-participants variability within a PHCC accounted for 89% of the total variation. Both glucose and oxidized LDL were positively associated with GSH-Px activity after adjustment for possible confounder variables (P = 0.03 and P = 0.01, respectively).

**Conclusion:**

In a population at high cardiovascular risk, a positive linear association was observed between plasma GSH-Px activity and both glucose and ox-LDL levels. The high GSH-Px activity observed when an oxidative stress situation occurred, such as hyperglycemia and lipid oxidative damage, could be interpreted as a healthy defensive response against oxidative injury in our cardiovascular risk population.

## Introduction

Coronary heart disease (CHD) is a major cause of morbidity and mortality in the developed world [1]. Atherosclerosis, characterized by the accumulation of cholesterol deposits in large and medium-sized arteries, is the most common pathologic process underlying cardiovascular disease and is often clinically manifested as coronary, cerebrovascular, and/or peripheral arterial disease [2]. An imbalance between antioxidant and oxidant-generating system that leads to oxidative stress has been proposed in the pathogenesis of atherosclerosis [2]. In particular, the oxidation of low density lipoproteins (LDL) by free radicals plays a central role in the formation, progression, and rupture of atherosclerotic plaques [2].

Mammalian cells are, however, protected from free radicals by a wide range of antioxidants such as the scavenger antioxidant enzymes [3]. Glutathione peroxidase is the general name for a family of multiple isozymes that catalyze the reduction of H_2_O_2_ or organic hydroperoxides to water or the corresponding alcohols using reduced glutathione (GSH) as an electron donor. In mammals, 8 glutathione peroxidases (GPx1–GPx8) have been identified to date, including both selenium-containing GPxs (GPx1–4 and 6) and their non-selenium congeners (GPx5, 7 and 8) [4].

Oxidative stress elicits an induction of antioxidant enzymes, as reported in a recent systematic review [5]. However, most studies were done in animal models; studies that have analyzed this association in humans have reported conflicting results [6]–[15].

The aim of the present study was to assess the association between the scavenger antioxidant enzyme glutathione peroxidase (GSH-Px) activity in plasma and novel and classical cardiovascular risk factors in elderly individuals at high risk for cardiovascular disease.

## Methods

### Study design

A cross-sectional study with baseline data from a subsample of the PREDIMED (PREvención con DIeta MEDiterránea) study was performed. The PREDIMED study is a large, parallel-group, multicenter, randomized, controlled, clinical trial aimed at assessing the effects of the traditional Mediterranean diet (TMD) on the primary prevention of cardiovascular disease (www.predimed.es and www.predimed.org). The PREDIMED detailed protocol of the study has been previously published [16].

### Subjects

Of the 7,447 participants aged 55 to 80 years from 8 Spanish PHCCs who were randomized to the PREDIMED study groups, 1,069 were randomly selected a posteriori for plasma measurements of glutathione peroxidase-1 activity and included in the present study. Inclusion criteria were the presence of diabetes or at least 3 CHD risk factors: current smoking; hypertension (systolic blood pressure ≥140 mm/Hg, diastolic blood pressure ≥90mm/Hg, or treatment with antihypertensive drugs); dyslipidemia (high-density lipoprotein [HDL] cholesterol <40 mg/dL for men and <50 mg/dL for women, LDL cholesterol>160 mg/dL, or treatment with cholesterol-lowering drugs); overweight or obesity (body mass index (BMI)>25 kg/m^2^), or family history of premature CHD. Exclusion criteria were history of cardiovascular disease, any severe chronic illness, drug or alcohol addiction, history of allergy or intolerance to olive oil or nuts, or low predicted likelihood of changing dietary habits according to the stages of change model. Individual eligibility was based on a screening visit by the primary care physician.

### Baseline assessments

The baseline examination included the administration of 3 types of questionnaire: 1) a validated food frequency questionnaire [17] and an assessment of the degree of adherence to the TMD, assigning a value of 0 or 1 to each of 14 questionnaire items [18], with energy and nutrient intake calculated from Spanish food composition tables [19]; 2) the Minnesota Leisure Time Physical Activity Questionnaire, which has been validated for its use in Spanish men and women [20],[21]; and 3) a 47-item general questionnaire assessing life-style, health conditions, smoking habits, sociodemographic variables, history of illness, and medication use. Weight and height were measured with calibrated scales and a wall-mounted stadiometer, respectively. Waist circumference was measured midway between the lowest rib and the iliac crest using an anthropometric tape. Trained personnel measured blood pressure in triplicate with a validated semi-automatic sphygmomanometer (Omron HEM-705CP, The Netherlands) with the patient in a seated position after a 5-minute rest.

### Laboratory analysis

Biological samples were obtained after an overnight fast, coded, shipped to central laboratories, and frozen at −80°C until the assay. Plasma glucose and lipid analyses were performed in a PENTRA-400 autoanalyzer (ABX-Horiba Diagnostics, Montpellier, France). Soluble HDL cholesterol was measured by an accelerator selective detergent method (ABX-Horiba Diagnostics, Montpellier, France) and LDL cholesterol was calculated by the Friedewald equation whenever triglycerides were <3.4 mmol/L. Quality control was performed with UNITY External Quality Assessment (BIO-RAD, Hercules, CA, USA). Circulating oxidized LDL (ox-LDL) plasma levels were measured by a commercial enzyme-linked immunoabsorbent assay (Mercodia AB, Uppsala, Sweden). Intra- and inter-assay coefficients of variation were 2.8% and 7.3%, respectively. Plasma GSH-Px activity (GSH-Px; EC 1.11.1.9) was measured by a Paglia and Valentine [22] modification method using cumene hydroperoxide (Ransel RS 505, Randox Laboratories, Crumlin, UK) as a glutathione oxidant. Intra- and inter-run imprecision were 3.6% and 5.43%, respectively.

### Statistical analyses

Participants were divided into quintiles of plasma GSH-Px concentration based on the sample distribution. We ensured that the result was not due to multiple comparisons, so we conducted the Holm adjustment [23],[24]. This method is just as simple and generally applicable as the Bonferroni method, but much more powerful [25],[26].

We applied multilevel statistical procedures [27] to investigate both the association between individual cardiovascular risk factors and GSH-Px and to what extent differences between PHCTs may account for any variation in the outcomes. We modeled individuals (level-1 units) as nested within 8 PHCTs (level-2 units). We modeled the continuous outcome (GSH-Px) using the multilevel linear regression to allow for within-center correlation, applying the Full Maximum likelihood method of estimation. Initially, we examined whether there was a variation between Spanish PHCTs in GSH-Px activity by fitting an unconditional model with no predictors at any level, only the intercept and random errors at the individual and PHCT levels. The second model estimated the effect of individual (level 1) covariates on the outcome and whether these effects varied by PHCT (i.e., we allowed for level-2 random effects). We confirmed the appropriateness of modeling continuous variables as linear (fractional polynomials method).

All statistical tests were 2-sided at the 5% significance level. Analyses were carried out using the HLM for Windows multilevel package, version 10.1, Stata/SE version 9.1 (Stata Corp.).

### Research ethics

The study followed the principals contained in the Helsinki Declaration and successive revisions and the standards of good clinical practice. The protocol was approved by the Committee on Clinical Research Ethics (CEIC) of the Institut d′Investigació en Atenció Primària (IDIAP) Jordi Gol, and participants signed an informed consent. Data confidentiality was guaranteed according to the pertinent laws of Spain (Ley Orgánica de Protection de Datos de Carácter Personal, 15/1999, December 1).

## Results

Of the 1,060 included participants, 577 (54.4%) were female. The mean age was 66.7 (SD: 8.0) years. GSH-Px activity showed a normal distribution. Plasma glucose and ox-LDL levels increased across GSH-Px quintiles (*P*<0.05). (Table 1).

**Table 1:** Participant characteristics by quintiles of serum glutathione peroxidase activity (U/L).

	Quintile 1	Quintile 2	Quintile 3	Quintile 4	Quintile 5	*P* value for trend*
	<507	507567	567–618	618–685	>685	
	(n = 213)	(n = 219)	(n = 212)	(n = 215)	(n = 210)	
Age (years)	67.1±9.2	66.2±8.8	67.5±7.9	66.6±6.7	66.3±7.1	1.000
Sex (female), *n* (%)	122 (57.1)	116 (52.5)	123 (58.6)	113 (53.3)	107 (50.7)	1.000
Current smokers, *n* (%)	46 (21.8)	48 (22.4)	46 (22.1)	37 (17.5)	30 (14.5)	1.000
Diabetes, *n* (%)	94 (44.3)	99 (45.6)	96 (45.7)	112 (52.8)	118 (56.5)	0.076
Hypertension, *n* (%)	170 (79.7)	177 (81.1)	172 (82.2)	180 (84.8)	160 (76.8)	1.000
Systolic blood pressure (mmHg)	156±61	159±61	153±20	158±54	157±62	1.000
Diastolic blood pressure (mmHg)	88±63	89±63	84±11	89±56	89±64	1.000
Medication use, *n* (%)						
Antihypertensive agents	153 (73.2)	154 (74.0)	151 (73.7)	156 (75.7)	139 (67.8)	1.000
Lipid-lowering agents	78 (37.3)	96 (46.2)	89 (43.4)	84 (40.8)	95 (46.6)	1.000
Insulin	13 (6.2)	14 (6.8)	7 (3.4)	21 (10.3)	17 (8.3)	1.000
Oral hypoglycemic agents	57 (27.1)	55 (26.4)	55 (26.8)	68 (33.2)	71 (34.6)	0.435
Aspirin or other antiplatelet agents	52 (24.8)	40 (19.1)	43 (20.7)	63 (30.4)	49 (19.8)	1.000
Waist circumference (cm)	107.6±88.8	104.3±62.9	101.6±53.7	97.8±10.3	98.3±11.1	0.468
EEPA leisure time (kcal/day)	224±181	237±195	277±250	273±253	267±240	0.216
Glucose (mg/dL)	117±34	118±35	119±34	125±40	126±39	0.042
Cholesterol (mg/dL)						
Total	203±30	205.8±32.3	209.2±31.2	210.8±35.6	207.5±35.0	0.480
High density lipoprotein (HDL)	50.4±10.5	50.7±10.0	53.2±10.0	52.6±10.7	52.3±10.2	0.288
Low density lipoprotein (LDL)	126±26	128±27	131±26	133±28	130±29	0.216
LDL/HDL cholesterol ratio	2.52±0.62	2.56±0.59	2.51±0.58	2.59±0.61	2.52±0.58	1.000
Triglycerides (mg/dL)	132±63	133±62	124±53	124±55	122±65	0.435
Oxidized LDL (U/L)	70±24	74±26	74±26	76±26	78±27	0.042

Abbreviations: EEPA, daily energy expenditure in leisure-time physical activity. Data are shown as mean ±SD, median (interquartile range), or percentage.

* *P* value for trend adjusted for multiple testing using Holm correction [24]. One-factor analysis of variance, nonparametric Kruskal Wallis test, and chi-square test were used as appropriate.

Table 2 shows the results of both the unconditional and the adjusted model for the association between GSH-Px and cardiovascular risk factors. From the results of the unconditional model, the GSH-Px mean value was 612 U/L (SE: 12 U/L), with a significant variation between PHCCs, ranging from 549 to 674 U/L (Variance = 1013.5; *P*<0.001). The variation in the mean level of GSH-Px was 15 times greater within PHCCs than between PHCCs (Variance = 15266.6; *P*<0.001). Thus, 93.8% of the variability in GSH-Px activity was between participants within centers rather than between PHCCs. The adjusted model (Table 2) showed a reduction in the mean GSH-Px value to 577 U/L (SE: 50 U/L), with a range of variation between PHCCs from 499 to 656 U/L. Results of this model showed that glucose and oxidized LDL were significantly positively associated with GSH-Px activity (*P*<0.05).

**Table 2:** Fixed and random parameters from a multilevel linear regression model of GSH-Px activity.

	Adjusted Beta coefficient^a^	Standard Error	*P* value
UNCONDITIONAL MODEL			
Fixed parameters			
Intercept	612	12.43	<0.001
Random parameters^b^	Estimate		P
PHCC-level Variance	1013.51		<0.001
Individual-level Variance	15266.61		
ADJUSTED MODEL			
Fixed parameters			
Intercept	577	50.07	<0.001
Glucose (mg/dl)	0.31	0.13	0.021
Sex (female vs. male)	−9.27	10.34	0.370
Age (years)	−0.53	0.54	0.330
EEPA leisure time (kcal/day)	0.02	0.02	0.251
Smoking (current smoking vs. nonsmoker)	−19.50	11.88	0.101
Insulin (treatment vs. no treatment)	24.66	18.17	0.175
Oral hypoglycemic agents (use vs. no use)	10.36	11.58	0.371
Oxidized LDL (mg/dL)	0.47	0.20	0.017
LDL/HDL cholesterol	2.23	8.23	0.786
Waist circumference (cm)	−0.10	0.08	0.224
Random parameters^b^	Estimate		*P*
PHCC-level Variance	1600.17		<0.001
Individual-level Variance	14640.03		

Abbreviations: EEPA, daily energy expenditure in leisure-time physical activity. PHCC, primary health care center.

a Adjusted β, regression coefficient of the association between each variable in the model and GSH-Px activity, controlling for the other variables in the model.

b Random parameters are multilevel measures of outcome variation. PHCC was considered as random.

## Discussion

This study assessed the association between GSH-Px activity and classical and novel cardiovascular risk factors in an elderly population with high cardiovascular risk. We identified a positive association between plasma GSH-Px activity and glucose and ox-LDL levels. The associations were moderately strong and linear, and persisted after adjustment for age, sex, and other possible confounders.

Although several studies have reported an association between cardiovascular risk factors and GSH-Px, they included small sample sizes and report contradictory results [6]–[15]. Furthermore, the results shown were either unadjusted [7],[8],[10]–[13] or adjusted only by age or sex [6],[9],[14],[15]. Whereas several studies examined the association between GSH-Px activity and diabetes and reported lower serum GSH-Px activity in patients with type 2 diabetes than in non-diabetic participants [6],[7],[14], other studies found increased GSH-Px activity in diabetic patients, compared to control group [9],[15]. Finally, one study found no significant difference in GSH-Px activity between three study groups (diabetic patients with and without hypertension and pre-diabetic patients) [8]. In other results, two of these studies reported a positive relationship between BMI and the antioxidant activity of GSH-Px [9],[12], another study found significantly lower erythrocyte GSH-Px activity in obese women compared to normal weight women [10], and one study found no significant differences in GSH-Px activity between patients with essential hypertension and age- and sex-matched healthy controls older than 65 years [13].

The biological oxidative effects of reactive oxygen species (ROS) on lipids, DNA, and proteins are controlled by a wide spectrum of exogenous antioxidant mechanisms, such as vitamins and phenolic compounds in diet, and also by endogenous antioxidants such as the scavenger enzymes, among them GSH-Px [28]. Hyperglycemia is a situation in which ROS are generated [29]. In turn, ROS production induces GSH-Px generation at DNA transcriptional level [30]. Thus, high GSH-Px activity may result from a preservation of the enzyme by a high antioxidant status (with low generation of ROS) or from increased GSH-Px production stimulated by ROS. Therefore, GSH-Px activity may serve as an indicator of the balance between oxidative status and the bioscavenging of ROS by antioxidants. In the present study we measured the *in vivo* ox-LDL as a marker of oxidative stress in order to examine the relationship between oxidative stress or damage and GSH-Px antioxidant enzyme activity. In our cardiovascular risk population, mean (± standard deviation) ox-LDL values (74±26 U/L) were higher than those obtained in a healthy population (49±22 U/L) using the same method and antibodies as in our study [31]. The positive linear relationship obtained between GSH-Px and both glucose and ox-LDL in our population would be compatible with enhanced production of GSH-Px when oxidative status is increased.

Induction of GSH-Px activity has been proposed as the mechanism by which preconditioning exerts protection in myocardial infarction [30]. In addition, overexpression of intracellular GSH-Px in transgenic animal models has shown to prevent postischemic free radical injury [32]. Cardiovascular risk factors present in our population, such as hypertension, diabetes, hyperlipidemia, and obesity, have been previously linked to oxidative stress and oxidative damage [33],[34]. In this population, the fact that a high GSH-Px activity is observed when an oxidative stress situation occurs, such as hyperglycemia and lipid oxidative damage, could be interpreted as a healthy defensive response against the oxidative injury.

In our study, 93.8% of the variability in GSH-Px activity was due to variability among participants within PHCCs rather than differences between PHCCs. After accounting for individual characteristics, the individual variability was reduced to 90.1%. It can hardly be expected that factors such as presence of genetic variants or selenium levels (a GSH-Px cofactor) influence GSH-Px activity [35]. In selenium-deficient patients, selenium supplementation increases enzymatic antioxidant activity such as GSH-Px and decreases lipid peroxidation [36]. The major sources of selenium are plant foods, meat, and seafood, but the selenium content of foods varies geographically depending on soil and water concentrations and the use of selenium-containing fertilizers [37]. Selenium intake in southern Spain has been reported to be above the Recommended Dietary Allowance (RDA) [38]. However, it is unknown whether selenium intake varies among Spanish populations and whether this could explain part of the variability found within PHCCs in our Spanish population.

The potential of selenoproteins such as selenium-containing GPxs (GPx1–4 and 6) to protect against oxidative stress led to the expectation that selenium would also be protective against type 2 diabetes and other cardiovascular risk factors. However, more recent findings form observational studies and randomized clinical trials have raised concerns that high selenium exposure may lead to type 2 diabetes, insulin resistance, or hyperlipidemia [39]. Additional evidence is needed to provide new insights into the role of selenium and of specific selenoproteins in human biology, especially to clarify the underlying mechanisms linking selenium to chronic disease endpoints. Further epidemiological studies and randomized clinical trials across populations with a different selenium status should be conducted to determine the causal effect of selenoproteins on the development of cardiovascular risk factors and diseases.

This study has strengths and limitations. A strength is its large sample size, geographically widespread within Spain. In addition, the study was carefully conducted using standardized protocols. The multilevel methodological approach allowed us to control for clustering within the PHCCs, thus assessing both the individual independent factors that may influence outcomes and whether there was variability in the plasma GSH-Px activity across PHCCs.

One of the limitations of our study is its cross-sectional design, which can identify associations but not causality. Moreover, cross-sectional studies are notably subject to confounding; for this reason, the analysis was adjusted for possible confounding factors. A large cross-sectional study such as this one contributes to the establishment of new hypotheses for large prospective studies and clinical trials. The relationship between selenoproteins such as GSH-Px and cardiovascular risk factors is undoubtedly complex. Future studies should genotype participants and investigate the potential interactions between genotype, selenium intake or status, and selenoproteins. Besides, they should explore whether other individual factors or contextual features (such as factors linked to geographical or environmental characteristics of PHCTs) may account for variation in the GSH-Px values.

Our study focused on an elderly population and cannot be extrapolated to a general population. Another potential limitation is that a survival bias could have underestimated the association between GSH-Px activity and cardiovascular risk factors. It is possible that subjects with diabetes, obesity, or hypertension and low GSH-Px activity may have died or developed a cardiovascular disease, and therefore could not be included in our study. Moreover, we could not take into account the duration and control of diabetes in our analyses because these data were not available.

In conclusion, a positive linear association was observed between plasma GSH-Px activity and glucose and ox-LDL levels in an elderly population with high cardiovascular risk. The high GSH-Px activity observed when an oxidative stress situation occurs, such as hyperglycemia and lipid oxidative damage, would be compatible with an increase in antioxidant defenses against oxidative injury in our cardiovascular risk population. Further epidemiological studies and randomized clinical trials are needed to assess the impact of selenoproteins on the development of cardiovascular risk factors and diseases and the cause-effect relationships between hyperglycemia, oxidative status, and GSH-Px in elderly populations at high cardiovascular risk.

## References

[1] Lopez AD, Mathers CD, Ezzati M, Jamison DT, Murray CJ (2006) Global and regional burden of disease and risk factors, 2001: systematic analysis of population health data. Lancet 367: 1747–1757. 16731270 10.1016/S0140-6736(06)68770-9

[2] Stocker R, Keaney JF Jr (2004) Role of oxidative modifications in atherosclerosis. Physiol Rev 84: 1381–1478. 15383655 10.1152/physrev.00047.2003

[3] Huang H, Mai W, Liu D, Hao Y, Tao J, et al (2008) The oxidation ratio of LDL: a predictor for coronary artery disease. Dis Markers 24: 341–349. 18688083 10.1155/2008/371314 PMC3850607

[4] Brigelius-Flohe R, Maiorino M (2013) Glutathione peroxidases. Biochim Biophys Acta 1830: 3289–3303. 23201771 10.1016/j.bbagen.2012.11.020

[5] Karunakaran U, Park KG (2013) A systematic review of oxidative stress and safety of antioxidants in diabetes: focus on islets and their defense. Diabetes Metab J 37: 106–112. 23641350 10.4093/dmj.2013.37.2.106 PMC3638220

[6] Kesavulu MM, Rao BK, Giri R, Vijaya J, Subramanyam G, et al (2001) Lipid peroxidation and antioxidant enzyme status in Type 2 diabetics with coronary heart disease. Diabetes Res Clin Pract 53: 33–39. 11378211 10.1016/s0168-8227(01)00238-8

[7] Likidlilid A, Patchanans N, Poldee S, Peerapatdit T (2007) Glutathione and glutathione peroxidase in type 1 diabetic patients. J Med Assoc Thai 90: 1759–1767. 17957916

[8] Bandeira SM, Guedes GS, da Fonseca LJ, Pires AS, Gelain DP, et al (2012) Characterization of blood oxidative stress in type 2 diabetes mellitus patients: increase in lipid peroxidation and SOD activity. Oxid Med Cell Longev 2012: 819310. 23259029 10.1155/2012/819310 PMC3509371

[9] Taheri E, Djalali M, Saedisomeolia A, Moghadam AM, Djazayeri A, et al (2012) The relationship between the activates of antioxidant enzymes in red blood cells and body mass index in Iranian type 2 diabetes and healthy subjects. J Diabetes Metab Disord 11: 3. 23497678 10.1186/2251-6581-11-3 PMC3581104

[10] Amirkhizi F, Siassi F, Djalali M, Shahraki SH (2014) Impaired enzymatic antioxidant defense in erythrocytes of women with general and abdominal obesity. Obes Res Clin Pract 8: e26–e34. 24548574 10.1016/j.orcp.2012.07.004

[11] Baez-Duarte BG, Mendoza-Carrera F, Garcia-Zapien A, Flores-Martinez SE, Sanchez-Corona J, et al (2014) Glutathione Peroxidase 3 Serum Levels and GPX3 Gene Polymorphisms in Subjects with Metabolic Syndrome. Arch Med Res 45: 375–382. 24819036 10.1016/j.arcmed.2014.05.001

[12] Ferro FE, de Sousa Lima VB, Soares NR, de Sousa Almondes KG, Pires LV, et al (2011) Parameters of metabolic syndrome and its relationship with zincemia and activities of superoxide dismutase and glutathione peroxidase in obese women. Biol Trace Elem Res 143: 787–793. 21210247 10.1007/s12011-010-8940-6

[13] Rybka J, Kupczyk D, Kedziora-Kornatowska K, Pawluk H, Czuczejko J, et al (2011) Age-related changes in an antioxidant defense system in elderly patients with essential hypertension compared with healthy controls. Redox Rep 16: 71–77. 21722415 10.1179/174329211X13002357050897 PMC6837520

[14] Harani H, Otmane A, Makrelouf M, Ouadahi N, Abdi A, et al (2012) [Preliminary evaluation of the antioxidant trace elements in an Algerian patient with type 2 diabetes: special role of manganese and chromium]. Ann Biol Clin (Paris) 70: 669–677. 23207812 10.1684/abc.2012.0763

[15] Likidlilid A, Patchanans N, Peerapatdit T, Sriratanasathavorn C (2010) Lipid peroxidation and antioxidant enzyme activities in erythrocytes of type 2 diabetic patients. J Med Assoc Thai 93: 682–693. 20572373

[16] Martinez-Gonzalez MA, Corella D, Salas-Salvado J, Ros E, Covas MI, et al (2012) Cohort profile: design and methods of the PREDIMED study. Int J Epidemiol 41: 377–385. 21172932 10.1093/ije/dyq250

[17] Martin-Moreno JM, Boyle P, Gorgojo L, Maisonneuve P, Fernandez-Rodriguez JC, et al (1993) Development and validation of a food frequency questionnaire in Spain. Int J Epidemiol 22: 512–519. 8359969 10.1093/ije/22.3.512

[18] Schroder H, Fito M, Estruch R, Martinez-Gonzalez MA, Corella D, et al (2011) A short screener is valid for assessing Mediterranean diet adherence among older Spanish men and women. J Nutr 141: 1140–1145. 21508208 10.3945/jn.110.135566

[19] Mataix J. (2003) Tabla de composición de alimentos [Food composition tables]. Granada: University of Granada.

[20] Elosua R, Marrugat J, Molina L, Pons S, Pujol E (1994) Validation of the Minnesota Leisure Time Physical Activity Questionnaire in Spanish men. The MARATHOM Investigators. Am J Epidemiol 139: 1197–1209. 8209878 10.1093/oxfordjournals.aje.a116966

[21] Elosua R, Garcia M, Aguilar A, Molina L, Covas MI, et al (2000) Validation of the Minnesota Leisure Time Physical Activity Questionnaire In Spanish Women. Investigators of the MARATDON Group. Med Sci Sports Exerc 32: 1431–1437. 10949009 10.1097/00005768-200008000-00011

[22] Paglia DE, Valentine WN (1967) Studies on the quantitative and qualitative characterization of erythrocyte glutathione peroxidase. J Lab Clin Med 70: 158–169. 6066618

[23] Aickin M, Gensler H (1996) Adjusting for multiple testing when reporting research results: the Bonferroni vs Holm methods. Am J Public Health 86: 726–728. 8629727 10.2105/ajph.86.5.726 PMC1380484

[24] Holm S (1979) A simple sequantially rejective multiple test procedure. Scand J Stat 6: 65–70.

[25] Levin B (1996) On the Holm, Simes, and Hochberg multiple test procedures. Am J Public Health 86: 628–629. 8629710 10.2105/ajph.86.5.628 PMC1380467

[26] Wright S (1992) Adjusted p-values for simultaneous inference. Biometrics 48: 1005–1013.

[27] Goldstein (2003) Multilevel Statistical Models. 3rd edition. London: Arnold.

[28] Gutteridge JM (1995) Lipid peroxidation and antioxidants as biomarkers of tissue damage. Clin Chem 41: 1819–1828. 7497639

[29] Ceriello A, Taboga C, Tonutti L, Quagliaro L, Piconi L, et al (2002) Evidence for an independent and cumulative effect of postprandial hypertriglyceridemia and hyperglycemia on endothelial dysfunction and oxidative stress generation: effects of short- and long-term simvastatin treatment. Circulation 106: 1211–1218. 12208795 10.1161/01.cir.0000027569.76671.a8

[30] Zhou X, Zhai X, Ashraf M (1996) Direct evidence that initial oxidative stress triggered by preconditioning contributes to second window of protection by endogenous antioxidant enzyme in myocytes. Circulation 93: 1177–1184. 8653839 10.1161/01.cir.93.6.1177

[31] Covas MI, Nyyssonen K, Poulsen HE, Kaikkonen J, Zunft HJ, et al (2006) The effect of polyphenols in olive oil on heart disease risk factors: a randomized trial. Ann Intern Med 145: 333–341. 16954359 10.7326/0003-4819-145-5-200609050-00006

[32] Mital R, Zhang W, Cai M, Huttinger ZM, Goodman LA, et al (2011) Antioxidant network expression abrogates oxidative posttranslational modifications in mice. Am J Physiol Heart Circ Physiol 300: H1960–H1970. ajpheart.01285.2010 [pii]. doi:10.1152/ajpheart.01285.2010 21335461 10.1152/ajpheart.01285.2010 PMC3094079

[33] Guxens M, Fito M, Martinez-Gonzalez MA, Salas-Salvado J, Estruch R, et al (2009) Hypertensive status and lipoprotein oxidation in an elderly population at high cardiovascular risk. Am J Hypertens 22: 68–73. 19008862 10.1038/ajh.2008.313

[34] Weinbrenner T, Cladellas M, Isabel CM, Fito M, Tomas M, et al (2003) High oxidative stress in patients with stable coronary heart disease. Atherosclerosis 168: 99–106. 12732392 10.1016/s0021-9150(03)00053-4

[35] Jablonska E, Gromadzinska J, Reszka E, Wasowicz W, Sobala W, et al (2009) Association between GPx1 Pro198Leu polymorphism, GPx1 activity and plasma selenium concentration in humans. Eur J Nutr 48: 383–386. 19415410 10.1007/s00394-009-0023-0

[36] Monget AL, Richard MJ, Cournot MP, Arnaud J, Galan P, et al (1996) Effect of 6 month supplementation with different combinations of an association of antioxidant nutrients on biochemical parameters and markers of the antioxidant defence system in the elderly. The Geriatrie/Min.Vit.Aox Network. Eur J Clin Nutr 50: 443–449. 8862480

[37] Shamberger RJ (1980) Selenium in the drinking water and cardiovascular disease. J Environ Pathol Toxicol 4: 305–308. 7007559

[38] Navarro-Alarcon M, Cabrera-Vique C (2008) Selenium in food and the human body: a review. Sci Total Environ 400: 115–141. 18657851 10.1016/j.scitotenv.2008.06.024

[39] Stranges S, Navas-Acien A, Rayman MP, Guallar E (2010) Selenium status and cardiometabolic health: state of the evidence. Nutr Metab Cardiovasc Dis 20: 754–760. 21094028 10.1016/j.numecd.2010.10.001

